# Parental satisfaction with neonatal intensive care units: a quantitative cross-sectional study

**DOI:** 10.1186/s12913-018-3854-7

**Published:** 2019-01-15

**Authors:** Inger Hilde Hagen, Valentina Cabral Iversen, Erik Nesset, Roderick Orner, Marit Følsvik Svindseth

**Affiliations:** 1 NTNU Norwegian University of Science and Technology, Postbox 1517, 6025 Aalesund, Norway; 2 St Olav’s University Hospital HF, Tiller District Psychiatric Center, Trondheim, Norway; 3 Norwegian of Science and Technology, Faculty of Medicine and Health Science, 7491 Trondheim, Norway; 4 College of Social Science. University of Lincoln, Brayford Pool, Lincoln, Lincolnshire LN6 7TS UK

**Keywords:** NICU, Parents, Satisfaction, Family-centred care

## Abstract

**Background:**

Patients and users experiences are useful for monitoring the quality of the hospital provisions and to improve health care delivery. Research results on associations between parental satisfaction and their socio-demographic status are inconclusive. We have also found a scarcity of research on the associations between parental satisfaction and standards of neonatal intensive care (NICU) services. We used the Neonatal Satisfaction Survey (NSS-8) to collect data to explore associations between parental satisfaction and socio-demographic variables and, associations between parents’ satisfaction and NICU care-services.

**Methods:**

A total of 568 parents from six different NICUs geographically dispersed in Norway completed the (NSS-8). All responses were rated and analysed using nonparametric analyses and logistic regression.

**Results:**

Support from families and friends is the most important sociodemographic area which links to reported levels of parental satisfaction. The most important areas for parents’ satisfaction with NICU care services include the decision making processes regarding the infant, respect and empathy from staff, and the continuity of treatment and care. Parents were least satisfied with how NICUs facilitate ongoing care for siblings, parents and infants during later stages of their hospital stay. Parents reported being in need of more guidance and training in meeting their child’s needs.

**Conclusion:**

To increase and sustain parents’ satisfaction with NICU care considerations should be given to separate elements of the total provision made for affected families. This study suggests that health personnel could address the needs of all family members as these evolve through phases of their stays in hospitals; be more attentive to parents with very preterm infants and parents with long NICU admissions; provide support to siblings; and give more attention to parents’ needs for continuity of care, follow-up, and information.

## Background

Patient and next of kin satisfaction surveys are an important and frequently used part of measuring the quality of health care [1, 2]. Parental experience is a crucial measure of service quality from the patients and parents’ perspective, and contributes to their overall satisfaction [3, 4]. These experiences might be useful for monitoring the quality of care in hospital wards and could point to ways of improving health care delivery [5].

The birth of an infant is challenging for all parents. Intense emotions and stress are particularly acute when an infant is born prematurely or with health problems and admitted to a neonatal intensive care unit (NICU). Parental stress is related to worries over the infant’s health, the infant’s outcome, and alterations to the parenting role, and feelings of grief concerning the loss of a fully healthy child are common [6–9]. Rocha et al. (2011) found that parents with a high level of stress are less satisfied with the care of the doctors. Additionally, mothers with high level of stress are satisfied with the attendance of the nursing team [10]. Studies show significant associations between parental satisfaction with health care in NICUs and their ability to give appropriate care for their child [11, 12]. Generally, higher satisfaction with health care is reported to yield better treatment compliance [1].

Family-centred care (FCC), defined as “[…] an inter-disciplinary, comprehensive, and holistic care of neonates and families with maintaining their respect and dignity” ([13]. p272), is necessary to promote the quality of NICU care. FCC is considered the gold-standard medical concept in the NICU [13] and is implemented in most units in the western world, but varies between countries and units. However, research shows that there still is a way to go before it has been rooted [14]. Evaluating parent satisfaction is important in NICU settings, and validated instruments based on FCC principles are recommended [13]. Research shows that FCC reduces stress and anxiety among parents [11, 15] and increases parental satisfaction by giving them the opportunity to participate in the child’s health [16].

A variety of factors are crucial when measuring satisfaction and quality of health care in NICUs. In a study from California, McCormick et al. (2008) measured mothers’ satisfaction and found that the main predictor of satisfaction with NICU care is the child’s health at the time of the interview. They also found that mothers’ education level, age, and ethnicity are significant predictors of satisfaction. Older, more educated, and white mothers were more satisfied with health care compared to non-white mothers and those with lower family income [17]. In contrast, a study from Canada revealed that mothers’ age and education level are not significantly associated with satisfaction scores [18]. Tsironi (2012) found that parents’ gender and duration of infants’ hospitalization are the most significant factors for parental satisfaction [19]. In their review, Butt et al. (2013) found that few studies have been performed to measure the factors related to parental satisfaction with NICU care, coinciding with the limited consensus over which parental or child demographic variables are correlated with satisfaction [20]. There is growing evidence that support from other NICU-parents and staff are important for parents and that organized support from peer-to-peer and NICU staff has been beneficial [21, 22]. There is, however, scant research on what support from family and friends means for parents.

The relationships between health personnel and patients are key factors in a parent’s satisfaction with care in NICU. Parents need information, continuity of care and health personnel listening to their needs [5, 23, 24]. Improving quality necessitates gathering data both from the parents’ experiences with different factors as well as the level of satisfaction with each factor [25].

The literature identifies a need for more research on which socio-demographic variables are associated with patient satisfaction and factors important for parents being satisfied with the neonatal health care services, including factors related to patient-reported experiences. This study can contribute to new knowledge about the factors that have an impact on satisfaction and give recommendations of how to improve health care services to increase parents’ satisfaction. Thus, this study aims to investigate:

- Associations between parental satisfaction and socio-demographic variables.
- Associations between parental satisfaction and neonatal intensive care services

## Methods

A multicentre prospective cohort study was conducted between September 2015 and October 2016. The design of the study is nearly similar to the design of a former study using the same data set [26]. However, there were some minor differences compared to Hagen et al. (2018) because other variables were tested in this study. In the former study, we aimed to statistically validate the Neonatal Satisfaction Survey (NSS-13), which included 67 questions, in six geographically spread Norwegian NICUs, and we estimated that a total of 450 answered questionnaires were necessary for a proper factor analysis. Therefore, we wanted each unit to collect approximately 100 completed questionnaires. From the factor analysis, a new questionnaire (NSS-8) was developed including 51 questions. Compared to NSS-13 there was a reduction in the number of questions.

### Study population and sampling strategy

Participants were Norwegian or English-speaking parents admitted to one of the six NICUs whose admissions lasted for more than two days to have a time basis for expressing their views. The infants’ gestation ages ranged from 24 to 42 weeks. The NICUs also admitted infants up to 3 months after birth. Parents whose children died while in the unit were excluded from this study. Our goal was to provide enough data to perform proper statistical analysis to validate the NSS-8. Due to reports from nurses collecting data, the parents that did not answer the survey were lost mostly due to administrative reasons and exclusion criteria, and we concluded that our sample was probably representative.

The first author contacted the head of the clinic in 12 NICUs and asked them to participate in the study. All the NICUs were organized quite similarly and shared the same philosophy of treatment and care. Due to the number of participants volunteering to participate and the fact that these hospitals also represent all geographical areas in Norway, we decided not to pursue contact with the six hospitals that did not answer the call for participation. The recruitment process followed a strategic selection according to the inclusion criteria. Participating NICUs varied in size from 6 to 21 beds (mean 12.5) and treated between 253 and 500 patients each year. Two NICUs are university hospitals, and the rest have regional or local catchment areas. Three units treated children of gestation age (GA) ≤ 23, while the rest provided care for children from GA 26–30.

### Data collection methods

The first author introduced the study to the unit nurses. Three research assistants in each hospital were responsible for questionnaire distribution and collection. During the data collection, the first author maintained regular contact with the research assistants via telephone and email. Some units were also visited during the data collection process. Research assistants in the participating NICUs identified parents who were eligible study participants. As discharge approached, the research assistant contacted the infants’ next of kin to secure their informed consent to take part in this study. The research assistant left a copy of the self-reported questionnaire with participating parents a few days before discharge from the unit. Parents with multiple births received only one questionnaire. To ensure confidentiality, parents dropped their completed survey form in a secured box at the unit.

### Measures

#### Socio-demographics

Mothers and fathers answered questions on demographic variables, such as age, level of education, native language, main income, civil status, and driving distance from home to hospital. Parents were also asked about their infant’s gestation age, number of children, and support from family and friends.

*A Neonatal Satisfaction Survey (NSS-8*) covering family-centred care principles [13, 14, 27] was used to gauge parental satisfaction with care-services provided within NICUs. The NSS-8 questionnaire contains 51 items and two overall satisfaction questions developed from literature reviews, focus group interviews with health personnel and parents of children in a neonatal unit. A pilot study was conducted [28]. Further validation was completed, and eight factors were extracted based on a principle component analysis of the 568 respondents [26]. These factors cover aspects related to *care and treatment*, *doctors*, *visits* (conditions, routines), *NICU facility*, *siblings* (facilitating for siblings), *information*, *parent anxiety*, and *discharge*. Cronbach’s alpha was calculated in the validation study [26] and varied between 0.70–0.94 for the eight factors. The factors are all validated and more thoroughly described in Hagen et al. (2018). The various questionnaire items were measured by a Likert scale with 5 alternatives. The NSS-8 is suitable for measuring parents’ overall satisfaction as well as their experiences in eight spheres of care. The questionnaire was translated from Norwegian to English and back to Norwegian.

### Ethics

The study was conducted according to the Helsinki Declaration. This project was first presented to the Regional Committees for Medical and Health Research Ethics, which reported that permission to conduct the project was not necessary (2015/386). The project was approved by the Norwegian Data Protection Authority. After having read an information letter concerning the study, all respondents were asked for oral and written consent to participate. We emphasized that participation was voluntary and that parents could withdraw from the study at any time.

### Analyses

The internal consistency of the NSS-8 was assessed in the main study using Cronbach’s α and item-total correlations of all 51 questions in NSS-8 [26]. Descriptive statistics, including frequencies and percentages, are shown for all eight NSS-8 factors and the socio-demographic variables. Continuous data were transformed into categorical data presented in descriptive statistics. Because data were strongly skewed (towards high satisfaction), nonparametric methods compared overall satisfaction scores and demographics, support, and single items in the NSS-8, as reported with descriptive values (median, range, mean, SD). Chi-square tests were used for analyses of associations between variables. The relationships among the eight factors of perceived satisfaction with care, demographic data, and support were investigated using Spearman’s rank correlations coefficient.

Because of the skewed dataset and to present our results in a readable crosstable, a nonparametric test was used, and the variables in NSS-8 were therefore dichotomized according to clinical decisions. The cut-off on the five-point Likert scale was set between those scoring “not at all”, “small degree”, and “some degree” of satisfaction (low satisfaction) and those scoring “largely” and a “very large extent” (high satisfaction). NSS-8 measures the degree of parental satisfaction with NICU health care-services. For the variable “stress, unrest and insomnia”, the cut-off was set to “not at all” to “in small degree” (low stress) and those scoring “some degree” to “very large extent” (high stress).

The two overall questions, *satisfaction with infant treatment* and *satisfaction with parent treatment* were dichotomized as follows: items 1–3 (very dissatisfied, quite dissatisfied, and neither satisfied or dissatisfied) were classified as “low satisfaction”. Items 4–5 (quite satisfied and very satisfied) were classified as “high satisfaction”. These two variables and selected dichotomized variables from the NSS-8 questionnaire were then cross tabulated.

Logistic regression was performed to assess the association between socio-demographic variables and support and the likelihood that respondents would report satisfaction with care. The dependent variable is a dichotomized version of the average of the 51 questions dealing with the different aspects of satisfaction, where (after clinical assessment) values from 1 to 4.1961 (the median value) were labelled as “low satisfaction” and values higher than 4.1961 as “high satisfaction”. The independent variables were categorical but the age of the parents was continuous in order to not lose power.

The questionnaire responses skewed markedly towards parents who were satisfied with NICUs. Aspects of care-services to be improved were difficult to establish by statistical means. Therefore, we decided to describe the areas where more than 10% (cut-off: *N* ≥ 14) of the respondents reported dissatisfaction with the NICU, given that they also reported dissatisfaction with the stay in the NICU in the overall question (Table 6). The 10% cut-off was used to exclude incidental responses, and on a desire to refrain from overanalysing dissatisfaction reports in such a skewed material. A two-tailed *p*-value less than 0.05 was considered statistically significant. All analyses were conducted via SPSS 25.

## Results

### Descriptive analyses

The response rate for the six participating hospitals varied from 33 to 66%, and the mean was 45%. Attrition analysis found that there were no differences between the non-responders families (*n* = 722) from those responding (*n* = 325) regarding the infant’s gestational age or length of stay, as shown in a table in a previous article [26]. The level of missing data in the completed forms was low (mean 1.1%), suggesting that the NSS-8 questionnaire is understandable and easy to answer.

Table 1 shows the descriptive statistics of the eight NSS-8 factors and selected background and socio-demographic variables. All eight factors had high mean scores, which is quite normal in such analyses. Of the 568 parents that completed the questionnaire, 312 (54%) were mothers and 256 (45%) were fathers. The mean age in the sample was 30 years of the mothers (SD 5.50) and 33 years for the fathers (SD 6.94). There was a significant difference in education between mothers and fathers (*p* = 0.013), where 184 mothers (59%) had a higher education (≥ 4 years) compared to 116 fathers (46%), and 95 mothers (30%) were undergoing unpaid work/education versus 24 fathers (9%).

**Table 1:** Descriptive statistics: NSS-8 factors and sociodemographic variables, parents (^a^)

	N	Median (Range)	MEAN (SD)
Factors in NSS-8			
F1 Care and treatment (1–5)	493	4.6 (2.0–5.0)	4.5 (0.5)
F2 Doctors (1–5)	547	4.1 (1.0–5.0)	4.0 (0.7)
F3 Visit (1–5)	543	4.3 (1.3–5.0)	4.4 (0.7)
F4 Information (1–5)	526	4.3 (1.0–5.0)	4.2 (0.7)
F5 Facility (1–5)	512	4.5 (1.8–5.0)	4.4 (0.6)
F6 Parent anxiety (1–5)	562	4.0 (1.0–5.0)	3.8 (0.9)
F7 Discharge (1–5)	549	4.0 (1.7–5.0)	4.0 (0.8)
F8 Siblings (1–5)	171	4.0 (1.0–5.0)	3.7 (1.1)
Total satisfaction of NSS-8	432 (76%)		
Demographic Variables			
Mother’s age	312	29 (18–42)	30.1 (5.5)
Father’s age	256	32 (19–62)	33.1 (7.0)
	N (%)		
Education level mothers:			
Higher ed. > 4 years	76 (24)		
Higher ed. < 4 years	108 (35)		
College	113 (36)		
Grammar school	15 (5)		
Education level fathers:			
Higher ed. > 4 years	50 (20)		
Higher ed. < 4 years	66 (26)		
College	126 (50)		
Grammar school	12 (4)		
Work status mothers:			
In paid work	217 (70)		
Not paid work/education	95 (30)		
Work status fathers:			
In paid work	232 (91)		
Not paid work/education	24 (9)		
Main income mothers:			
In paid work	233 (75)		
Not in paid work	79 (25)		
Main income fathers:			
In paid work	236 (92)		
Not in paid work	20 (8)		
Marital status mothers:			
Married/in relationship	300 (96)		
Divorced/single parent	11 (4)		
Marital status fathers:			
Married/in relationship	247 (97)		
Divorced/single parent	7 (3)		
Language mothers:			
Norwegian	275 (88)		
Not Norwegian	37 (12)		
Language fathers:			
Norwegian	232 (91)		
Not Norwegian	24 (9)		
Travel time to hospital mothers:			
Less than 1 h	156 (50)		
More than 1 h	156 (50)		
Travel time to hospital fathers:			
Less than 1 h	137 (54)		
More than 1 h	119 (46)		
Length of stay			
2 days – 2 weeks	347 (61)		
> 2 weeks	221 (39)		
Sole provider mothers:			
Yes	124 (40)		
No	188 (60)		
Sole provider fathers:			
Yes	25 (10)		
No	231 (90)		
Support from family and friends mothers:			
Support	275 (88)		
Not support	37 (12)		
Support from family and friends fathers:			
Support	232 (91)		
Not support	24 (9)		

Median and range reported as appropriate for non-parametric data and Mean and SD are reported as additional data information

(^a^) Partly reported in Hagen [26] due to same data collection

A total of 275 (88%) mothers reported receiving support from family and friends compared to 232 fathers (91%). A total of 124 mothers (40%) reported being alone with the infant in the NICU versus 25 fathers (10%). Most of the parents were Norwegian (*N* = 275, 88% mothers; 232, 91% fathers), and the length of the NICU stay ranged from two days to two weeks (median = 2 weeks; SD, 1.078). Most of the parents (532, 94%) characterized their child’s health as good, while 22 (4%) characterized their child’s health as poor (Table 1).

Of the 352 infants in the study, 245 (70%) were born with GA ≤ 37. There were 29 couples with twins (Table 2).

**Table 2:** Demographics of the parents’ infant (*N* = 352) participating in the study. (^a^)

Variables	Total (%)
Was your child premature or born at term?	
Premature (< 37 weeks)	245 (70)
Born at term (≥ 37)	107 (29)
Multiple birth	29 (1.0)
Parents’ evaluation of the child’s health (N = 568)	
Good	532 (94)
Poor	22 (4)
Missing	14 (2)

(^a^) Partly reported in Hagen [26] due to same data collection

Parental satisfaction items were skewed towards positive assessment (mean 4.15, SD .46) on a scale of 1–5, where 5 represents the most positive score. A total of 432 (76%) were highly satisfied with NICUs, answering from “largely” to “a very large extent”. For the two overall questions, we gathered a total score for the parent’s satisfaction with the care of the infant. In the first question, 99% reported satisfaction; in the second question (parents’ satisfaction with other elements of care), 91% of the parents reported satisfaction.

Cronbach’s alpha for the eight factors in NSS-8 was the same as in our previous article as follows: factor 1; *care and treatment* 0.94, factor 2; *doctors* 0.91, factor 3; *visits* 0.91, factor 4; *information* 0.81, factor 5; *facility* 0.72, factor 6; *parental anxiety* 0.74, factor 7; *discharge* 0.70, and factor 8; *siblings* 0.72. The item-total correlation was 0.95. Corrected item-total correlation showed that none of the single items in the questionnaire was higher than the item-total correlation, which indicates that each item correlates well with the total score [26].

### Correlations between NSS-8 factors and demographics and support

In the correlation matrix (Table 3), there were small to moderate significant correlations between seven out of thirteen independent areas (gender, education level, duration of stay, support, infants’ health, GA, and single/multiple birth) and the eight NSS-8 factors. Parents’ age, language, main income, travel time, civil status, and sole providers were not statistically correlated with any of the eight NSS-8 factors. Mothers were more anxious compared to fathers and parents’ education level was negatively correlated with *doctors*, indicating that those with less education were more likely to be satisfied with NICU doctors. The duration of stay showed a significantly negative correlation with *doctors*, *facility*, and *parental anxiety*. In other words, a longer duration in the NICU decreased satisfaction with *doctors* and the *facility* and increases anxiety.

**Table 3:** Significant Spearman’s rank correlations^a^ between NSS-8 and demographic data, support and child’s health. Total *N* = 568

Demografic	Care and treatment	Doctors	Facilities	Information	Visit	Parents anxiety	Discharge	Siblings
Gender						.100*		
Parent age								
Language								
Education level		−.090*						
Main income								
Marital status								
Travel time								
Duration of stay		−.102*	−.213**			−.257**		
Sole provider								
Support from family and friends	.338**	.268**	.128**	.303**	.242**	.119**	.249**	
Infant health	.195**	.121**	.157**	.134**	.138**	.318**	.227**	.180*
Gestation age	.100*	.154**	.268**		109*	.162**		
Single or multiple birth		−.112**			−.087*			

*: *p* ≤ 0.05. **: *p* ≤ 0.01 (2-tailed)

^a^Non-significant correlations are excluded

Receiving support from family and friends and infants’ health were the area’s most important for satisfaction level. A higher level of support increased the satisfaction level and decreased parents’ anxiety. Infants’ health was significantly and positively correlated with all eight factors, indicating that better infant health led to greater satisfaction with the NICU and less parental anxiety.

Parental satisfaction with the factors *care and treatment*, *doctors*, *facility*, and *visits* in NICU increased with higher GA. Parental anxiety indicated that the later gestation, the less fear among parents concerning the infant, although the effect size was very small (16%). Single or multiple birth was significantly and negatively correlated with the factors *doctors* and *visits.*

### Associations between total satisfaction (NSS-8) and socio-demographics and support

The logistic model that included all independent variables showed a significant improvement compared to the base model with only the constant term, as indicated by the chi-square test of the change in log-likelihood (χ^2^ (13, *N* = 568) = 65.356, *p* < 0.01). According to Table 4, the model explained between 15% (Cox and Snell R square) and 20% (Nagelkerke R squared) of the variance in satisfaction status. Our model showed an overall classification accuracy rate of 65.7%, which is more than 25% higher than the proportional-by-chance accuracy rate of 50%.

**Table 4:** Logistic regression predicting likelihood of reporting high and low satisfaction with NICU

	B	S.E.	Wald	P	Odds ratio
Gender	−.293	.258	1.287	.257	.746
Parent age	.065	.020	10.158	.001	1.067
Language	−.355	.464	.584	.445	.702
Education level	−.646	.234	7.620	.006	.524
Main income	−.261	.301	.749	.387	.771
Marital status	−.836	.717	1.361	.243	.433
Travel time	−.062	.090	.485	.486	.940
Duration of stay	−.074	.155	.230	.632	.929
Sole provider	−.308	.278	1.223	.269	.735
Support	.867	.174	24.681	< 0.001	2.379
Infant health	−.560	.279	4.014	.045	.571
GA	.327	.158	4.258	.039	1.387
Single or multiple birth	−.381	.401	.901	.342	.683
Constant	−5.670	1.287	19.414	.000	.003

Cox and Snell R square 15%, Nagelkerke R squared 20%

X ^2^ (13, N = 568) =65.356, p < 0.01

Parents’ age, education level, support from friends and family, infants’ health and infants’ GA made a unique statistically significant contribution to the model. The most important area was support from family and friends. The model indicated that a parent with support from family and friends has an odds of being satisfied with the NICU that is 2.4 times that of a parent with no such support. The second most important area was infant gestation age. A Parent to an infant born at term has an odds of being satisfied that is 1.4 times that of a parent to an infant born extremely premature. The third most important area is parents’ age. Older parents has an odds of being satisfied that is 1.07 times higher compared to younger parents. A parent reporting good infant health has an odds of being satisfied that is only 0.57 times that of a parent reporting bad infant health. Finally, a parent with primary or high school has an odds of being satisfied that is 0.52 times that of a parent with college or university education (controlling for all other factors in the model).

### Associations between parental satisfaction and neonatal intensive care services

Parents who had one doctor with the principal responsibility for the child were significantly more satisfied with the NICU than those not experiencing such continuity. The same was found with parents reporting a permanent group of caregivers looking after the infant compared to those not experiencing continuous support. Those reporting that care personnel had time for parents were also significantly more satisfied with NICU. Similarly, perceiving respect and understanding from health personnel led to significantly greater satisfaction (Table 5).

**Table 5:** Associations between perceived high and low satisfaction and some of the clinical interesting items from NSS-8

	Overall question about parents satisfaction with care of the infant		p	ES (Phi)
	Low satisfaction N (%)	High satisfaction N (%)		
One doctor responsible (N559)				
Low degree	30 (77)	307 (59)	.042	.09
High degree	9 (23)	213 (41)		
	Overall question about parent satisfaction with care of parents		p	ES (Phi)
	Low satisfaction N (%)	High satisfaction N (%)		
Continuity of care (N564)				
Low degree	22 (46)	135 (26)	.006	.12
High degree	26 (54)	381 (74)		
Care personnel signaled that they had time for parents (N560)				
Low degree	16 (33)	25 (5)	< 0.001	.31
High degree	32 (67)	487 (95)		
Personnel showed understanding and respect for parents situation (N562)				
Low degree	15 (31)	22 (4)	< 0.001	.30
High degree	33 (69)	492 (96)		
Consideration and care from nurses (N561)				
Low degree	21 (44)	59 (11)	< 0.001	.26
High degree	27 (56)	451 (88)		
Consideration and care from doctors (N556)				
Low degree	27 (57)	150 (29)	< 0.001	.17
High degree	20 (43)	359 (71)		
Care personnel were interested in hearing your opinions as parents (N560)				
Low degree	16 (33)	61 (12)	< 0.001	.17
High degree	32 (68)	451 (88)		
Doctors were interested in hearing your opinions as parents (N557)				
Low degree	23 (48)	131 (26)	.002	.14
High degree	25 (52)	378 (74)		
Stress (N560)				
Low degree	19 (40)	263 (51)		
High degree	29 (60)	249 (49)	.16	.07

Effect size (Phi) = small effec = .10, medium = .30 and large = .50

There were also significant associations between parents reporting the perceived consideration and care from nurses and doctors and satisfaction with care. Moreover, there was a significantly positive association between satisfaction and having health personnel who were interested in listening to parents’ opinions on treatment and care for the infant.

Finally, questions about parental stress, unrest, and insomnia in connection with the NICU stay had a mean score of 3.5 (SD 1.1), indicating a large degree of perceived stress. We therefore wanted to investigate if perceived stress was associated with parental satisfaction for the entire NSS-8, which was not significant.

Table 6 shows the distribution of respondents who reported dissatisfaction with NICU care-services and questions with the highest frequency of dissatisfaction. Parents were most dissatisfied with how NICUs are prepared for the infants’ siblings. Improvements were also needed in the following areas: continuity of care, information, and follow-up.

**Table 6:** Potential for improvement: Parents’ perception of dissatisfaction with NICUs services. N 136

N	Questions from NSS-8
Overall question about parents satisfaction with care of the infant	
30	To what extent did you experience that one doctor had the principal responsibility for the child?
Overall question of parents satisfaction with their one care	
35	To what extent were the siblings’ reactions paid attention to?
35	To what extent are you satisfied with the activities offered to the child’s siblings?
29	To what extent did you experience stress/anxiety/sleeplessness in connection with the stay at the unit?
28	During the child’s admission, do you think you were given the necessary information about the effects and side effects of new medication given to the child?
27	To what degree do you think the doctors showed care and consideration for the child?
26	Have you been given information about what to do if the child become ill/have a relapsed/need medical attention after retuning home?
25	To what degree do you think the doctors signaled that they had time for you?
24	While the child were admitted, were you at any time afraid that the child would have delayed injury/after-effects?
23	To what extent did you experience that the care personnel provided relief or assistance to the admitted child during the stay?
23	To what degree do you think the doctors were interested in hearing your opinions as next of kin?
22	To what extent did you experience that you were taken care of later in the process?
21	To what degree do you think the doctors appeared professionally competent?
21	To what extent did you experience that the care personnel had consideration and care for you?
17	Do you think you were given the necessary information for the period following discharge?
17	To what degree do you think the doctors gave you and your child sufficient information regarding the prognosis/outcome?
17	To what extent did you experience that you receive guidance /training in meeting your child’s needs?
16	To what extent did you experience that the care personnel were interested in hearing your opinion as a next of kin?
16	To what extent did you experience that the care personnel signaled that they had time for you?
16	Were you angry, upset or disappointed in the hospital personnel during the stay?
16	Do you think you were given the necessary information about how tests and examinations were to be carried out when the child were admitted?
15	To what extent did you experience that you were taken care of upon arrival at the unit?
15	I experienced that the personnel showed understanding and respect for our situation
15	To what extent did you experience that the care personnel took your family situation into consideration?

N = those parents (> 10%) that reportede low satisfaction with items and low satisfaction with overall item

## Discussion

We conducted a cross-sectional study to investigate associations between parental satisfaction and socio-demographic variables and, associations between parents’ satisfaction and neonatal intensive care-services.

Most parents reported moderate to high levels of satisfaction with NICUs (76%). High satisfaction levels were also observed internationally [17, 20, 24] and in other health care units in Norway and in comparable countries [29, 30]. FCC is the standard in NICUs, and parents are encouraged to spend more time with their infants and to participate in their care. Research has shown that FCC can contribute to improving satisfaction and reducing distress among parents [31, 32].

Regarding socio-demographic variables, the study found that support from families and friends, followed by infants’ gestation age, parents’ age, infant health, and parents’ education level were the most important areas for satisfaction.

Except for the factor *siblings*, support from family and friends was statistically positive and significantly associated with all NSS-8 factors. The regression model also unearthed support as the most crucial question, indicating that when controlling for all other demographic questions in the model, parents receiving support from family and friends were 2.4 times more satisfied as a whole with the NICU than those lacking support. To our knowledge, no other studies have explored the association between satisfaction with the NICU and support from family and friends. One study, however, explored patient satisfaction with the health-care system and concluded that patient satisfaction depends more on areas external to the health system compared to the experience of care as a patient [29]. Some studies have pointed to family and friend involvement as a coping strategy [33, 34], which is consistent with FCC principles [35].

In our study, infants’ gestation age was significantly and positively related to five of the eight NSS-8 factors, indicating that the closer to term the baby is born, the more satisfied parents. Our regression analysis also indicated that gestation age was the second most important variable when controlling for all other demographic questions. One study from the USA found that parents of infants with a gestational age ≤ 32 weeks were significantly more likely to report feeling confused compared to parents of less premature infants [36]. Confusion and lack of control can often lead to dissatisfaction. However, a previous review study did not find similar correlations, and report ambiguous findings when comparing birth weights and satisfaction [20].

Parents’ age was not significant for any of the eight factors in our correlation matrix. Nevertheless, we found that parents’ age was the third most important areas and was positively and significantly related to total satisfaction with NICU, indicating that older parents were more satisfied. This result is consistent with one other study [17], although this association was not reported in other studies. Wong et al. (2011) found that age was not significantly related to parental satisfaction, while Tsironi et al. (2011) found that younger parents were significantly more satisfied than older parents were.

In our study, education level emerged as negatively and significantly related to satisfaction with one of the NSS-8 factors, namely *doctors*. The regression analysis also revealed a negative and significant relation to the total score of satisfaction, indicating that those with lower education were more satisfied with the NICU. This result is consistent with Tsironi’s study [19], who also found that parents with basic education expressed a higher level of satisfaction, possibly explained by their lower expectations and demands from the health care system. A Canadian study found no association between parental education level and parental satisfaction [18].

For all NSS-8 factors in the correlation matrix, infant health as rated by the parents was also negatively and statistically associated with parental satisfaction. The regression model revealed that parents of infants in good health were more satisfied with the NICU compared to those who did not rate their infant’s health as good. A previous study found that the major predictor of parental satisfaction with neonatal intensive care was infant health at the time of the interview [17]. A review article found little consensus between satisfaction with the NICU and infant or parental demographic variables [20]. Similar to our study, however, they found that some studies pointed to a positive association between satisfaction ratings and parental perceptions of their infant’s health.

The amount of variance explained by the variables in our study was small, although this is similar to other studies [17]. On the other hand, support from family and friends explained the largest share of variance in satisfaction. The implications for praxis is that health personnel, when caring for parents who lack good relations with family and friends, must keep in mind the strong and positive association between parental satisfaction and support from family and friends.

Being a parent to a premature or sick newborn infant who is admitted to the NICU is well documented as a stressful event [7, 9, 37]. We found no significant relationship between perceived stress and satisfaction. A Portuguese study found that mothers’ stress levels increased when they were not satisfied with doctors [10], although cultural differences can make comparability between Portugal and Norway difficult. We have not found other studies investigating if satisfaction is impacted by parental stress. In our correlation matrix, gender was positively and significantly related to parental anxiety, indicating that compared to fathers, mothers were more stressed and anxious about the health and well-being of their child. Moreover, even if parents report a high level of satisfaction with the NICU, they might also experience high levels of stress at the same time. It is, however, reasonable to believe that high levels of stress may decrease tolerance to environment, which again could influence satisfaction level. Parents will worry about their child’s health and well-being, and health personnel cannot always succeed in treatment, nor can they always promise that everything will be fine.

The most important areas for parents’ satisfaction with NICU care-services were involvement in decision making regarding the infant, respect and empathy from staff, and continuity of treatment and care. It is tempting to believe that if parents will be able to make decisions for the infants’ treatment and care, it will be important for them to have a good relationship with the NICUs doctors and nurses. This is in consistence with a review study were they found that important areas for making decisions for their infants are the perceptions of communication and relationships with the health personnel [38].

In the present study, we also found that the relationship between health personnel and parents is an important area for parental satisfaction with NICU. We found significantly greater satisfaction among those parents who reported that one doctor had responsibility for the child, that they had one permanent group of caregivers, and when health care personnel had time for parents and conveyed respect and understanding.

Other studies have pointed out the relationship between patient and practitioner as the most important health service area affecting patient satisfaction [12, 39–45]. This emerges as a key area in parents’ satisfaction with care in the NICU [5, 23, 24]. The FCC statements also highlight this relationship as important when caring for infants in hospitals [35].

Approximately one-fourth of parents in our study showed moderate to low satisfaction. However, room for improvement may be found, even when a service is regarded as good or excellent. Questionnaire responses reflect a high level of quality for the full range of NICU care-services and as such sets a baseline to aspire to. The study revealed some specific areas on which health personnel should focus. The worst performance was supporting the infants’ siblings, which is an integral part of assuring high-quality services under the FCC approach in NICUs. Unfortunately, and despite the efforts made to support siblings, there are too few studies on sibling support and comprehensive services [46].

The present study, along with other studies [23, 24, 42], demonstrate that parents need health care personnel to provide consideration, information, and continuity of care during the entire period in the NICU. These findings convey that health care professionals have an opportunity to increase parental satisfaction in the NICU and help to improve outcomes.

### Limitations

In the present study, parents answered the NSS-8 just before discharge from the NICU. We assume that at this time, parents are often more satisfied than they would have been earlier in the process or immediately after discharge. Just before discharge, parents probably experience a stabilized situation. They are often familiar with the health personnel; they manage to care for the infant in a familiar and safe atmosphere closely watched by NICU experts. The infant’s health is acceptable or good, and the parents often look forward to taking the new family member home to the rest of the family. Hence, the timing of the NSS-8 may have skewed the results towards greater satisfaction. Furthermore, this positive bias might not be reduced when introducing the survey earlier in the NICU stay. Indeed, a positive bias might result from the possible unwillingness of respondents to answer negatively during the stay. We believe that this unwillingness is less important shortly before discharge. Using text-message questions sent to the parents mobile phones during the NICU stay [47] could be used as an alternative to a questionnaire to measure parents’ satisfaction with the neonatal care and perhaps this could influence the response bias.

Another limitation is that the amount of variance explained by the study’s variables was quite modest, although this modesty was reflected in other studies [17]. Finally, the total number of admissions in the period of gathering data was 1175 new-borns. The exclusion criteria and administrative challenges were the main reasons for not answering the survey. Attrition analyses were performed for the variables *length of stay* and *gestation age,* and we found congruence between the sample and the total population. In our NSS-8 the demographic question about the infant gestation age is from 24 week to 42 week. Today it is a consensus of try to rescue infant from > = 23 weeks and we will change this in our next version.

## Conclusions

The NSS-8 is a suitable tool for monitoring and spotting early stages of declining service standards, helping to identify specific questions that contribute to service decline.

An understanding of what is satisfying to parents would help to identify areas of caregiving in need of change and to decide which interventions to implement to further support families. In summary, this study expands the rather limited literature on areas associated with parental experiences and satisfaction during admission to the NICU. Giving birth to a preterm or sick infant is a distressing and traumatic time for most parents. Despite this, the present study suggests that parents are very satisfied with the treatment and care provided during the NICU stay. However, some elements need to be considered to increase and maintain satisfaction: be aware of parents who lack a good friend and family network; be more attentive to parents with very preterm infants and parents with longer NICU stays; provide support to siblings; and give greater attention to parents’ needs for continuity of care, follow-up, and information. Due to the response rate, the geographical spread of the hospitals and the statistical validation of the survey, the generalizability of the study is rather strong in Scandinavian settings.

The NSS-8 could possible also be used to compare satisfaction between units and countries, and monitor changes over time.

## Abbreviations

FCC: Family-Centred Care
GA: Gestation age
NICU: Neonatal Intensive Care Unit
NSS-8: Neonatal Satisfaction Survey 8
